# Observation of Coherent Perfect Absorption in Oil Film on Water Surface and Sensitive Detection of Refractive Index Anisotropy in the Film

**DOI:** 10.1021/acs.langmuir.3c01189

**Published:** 2023-08-01

**Authors:** Mayu Hasegawa, Junpei Oi, Kyohei Yamashita, Keisuke Seto, Takayoshi Kobayashi, Eiji Tokunaga

**Affiliations:** † Department of Physics, Faculty of Science, Tokyo University of Science, 1-3 Kagurazaka, Shinjuku-ku, Tokyo 162-8601, Japan; ‡ Advanced Ultrafast Laser Research Center, The University of Electro-Communications, 1-5-1 Chofugaoka, Chofu, Tokyo 182-8585, Japan; § Department of Electrophysics, National Yang Ming Chiao Tung University, Hsinchu 300, Taiwan

## Abstract

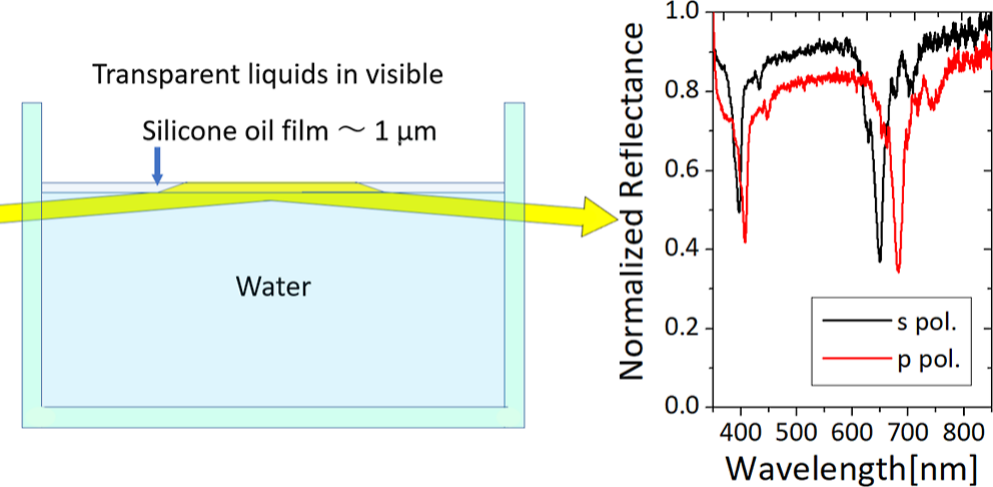

Sharp reflection dips of 50% were observed when white light was incident from the side of a cell on a 1 μm thick film of silicone oil (polydimethylsiloxane, PDMS, nearly transparent in visible light, with the extinction coefficient κ ≈ 0.0001) above a water surface in the cell so that the total reflection condition was satisfied at the oil-air interface. This is the first observation of a coherent perfect absorption (CPA) phenomenon in liquid. The experimental results can be reproduced by the Fresnel reflectance of the monolayer film, but the wavelength positions at which the dip appears for s-polarized and p-polarized light are reversed if the refractive index of the oil film is assumed to be isotropic. The experimental results were correctly reproduced by assuming that the extraordinary-ray refractive index (light polarized perpendicular to the interface) is 1% larger than the ordinary-ray refractive index (light polarized parallel to the interface). This indicates that the polarization dependence of the CPA phenomenon is extremely sensitive to the difference between the in-plane and out-of-plane refractive indices of the thin film.

## Introduction

Coherent perfect absorption (CPA) is a phenomenon in which 100% absorption is achieved by enhancing absorption through interference of coherent optical beams in a Fabry–Perot resonator. It was reported by Stone et al. in 2010.^1,2^ If two opposing beams are incident into a normally passive device, there will be two outgoing beams due to reflection and transmission, but in CPA, the energy is fully supplied to the system and there is no outgoing beam. Subsequently, realizations in various systems were reported.^3^ In the first proposal, two opposing coherent beams were incident into the sample, and by properly selecting the relative phase of the two coherent beams at the same wavelength, the energy was fully supplied to the system with perfect absorption at a wavelength that meets the CPA requirements.^1^ CPA thus realized requires a coherent laser source and delicate alignment of multiple laser beams, which does not make CPA readily usable for a wider range of applications. In contrast, a method in which a single beam is incident from one side of a sample with a highly reflective structure to obtain perfect absorption at wavelengths that satisfy the CPA conditions is called single-channel CPA (SCCPA). SCCPA does not necessarily require a coherent laser source and the delicate alignment of multiple laser beams. We have recently proposed and realized a simple method to achieve CPA with a single-beam incidence.^4^ We derived the conditions for a sample in which CPA is realized by simply an incoherent white light beam incident on a transparent substrate with a nearly transparent thin film deposited on it to satisfy the total reflection condition at the interface between the film and the surrounding medium and observed a CPA dip of over 90% in a polyvinylpyrrolidone (PVP) thin film on a MgF_2_ substrate. This is the simplest system that realizes SCCPA. This is also the simplest system that satisfies the critical coupling condition (the radiative loss rate equal to the non-radiative loss due to optical absorption in the medium) known prior to the report on CPA in 2010.^5−7^ Surface plasmon polariton (SPP) resonance observed with a thin metal film deposited on the hypotenuse surface of a right-angle prism by the attenuated total reflection (ATR) method is also a system on the same principle and is useful as an interface sensor that detects changes in the refractive index of an interface with high sensitivity.^8−10^ Similarly, waveguide mode sensors with transparent dielectric layers on prisms are based on the same principle.^11−13^ A review of recent progress in SCCPA has been reported as a method for realizing optical absorbers based on lossy films with thicknesses considerably smaller than the wavelength of the incident light.^14^ It is theoretically proposed that perfect absorption can be realized even for atomic monolayer materials such as graphene.^15−17^

In this paper, as noticed in future prospects in the preceding paper,^4^ we report on an experiment aimed at observing CPA in a transparent liquid thin film. We attempted to observe SCCPA in silicone oil (κ ∼ 0.0001), which has an order of magnitude smaller extinction coefficient than the polymer (PVP, κ ∼ 0.001) used in the preceding paper. At the same time, very interestingly, we also report that the s- and p-polarization dependence of the SCCPA spectrum allows us to evaluate the anisotropy of the in-plane and out-of-plane refractive indices of the oil film.

## Principles

**Figure 1:**
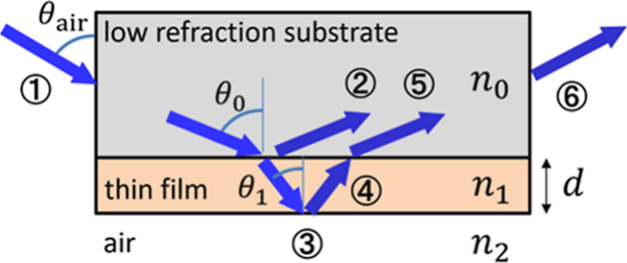
Mechanism of SCCPA using total reflection of a monolayer film with complex refractive index *n*_1_ sandwiched between media with refractive indices *n*_0_ and *n*_2_.

A thin film is sandwiched between a substrate and air (the surrounding medium), forming a Fabry–Perot resonator, and it is necessary to obtain as deep a dip as possible. To achieve this, it is known that the thin film material must have a high refractive index and the substrate material must have a low refractive index. In addition, the attenuation due to the absorption of the thin film material and the interfacial reflectance must meet a certain condition so that the surface reflected light and the multiply reflected light cancel each other out. In other words, CPA occurs in this experiment, as shown in Figure 1.

Collimated white lamp light is incident on the side of the substrate (①) under conditions where the angle of incidence θ_0_ from the substrate to the thin film is close to 90°. Light entering the substrate is reflected at the substrate–film interface (②) and incident on the film at the refracted angle θ_1_. Light entering the film undergoes total internal reflection (③) at the boundary with the air, and multiple reflections occur (④) (resonator) due to more than 90% reflectance at the boundary with the substrate. The light emitted from the thin film (⑤) has the same amplitude and opposite phase as the light reflected from the surface (②), and they cancel each other out due to destructive interference. Finally, the light emitted from the opposite side of the substrate (⑥) has a reflection dip with sharp absorption at a specific wavelength (a transmission dip if regarded as transmission from ① to ⑥). This is SCCPA using total reflection.

Let the substrate on the incident side, the thin film (thickness *d*), and the surrounding medium be labeled 0, 1, and 2, respectively, and let the respective refractive indices be *n*_0_ (real), *n*_1_ = *n* + *i*κ, and *n*_2_ (real), the angle of incidence from the substrate to the thin film be θ_0_, and the refracted angles in the thin film and in the surrounding medium be θ_1_ and θ_2_. The refractive indices and angles are related through Snell’s law as
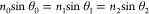
1Then, the complex amplitude reflection coefficient^18^ is given by
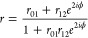
2where *k* is the wavenumber of the incident light in vacuum and ϕ = *kn*_1_*d* cos θ_1_ = *p* + *iq*. Therefore, the necessary condition for CPA is^14^
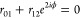
3

If the angle of incidence from the *n*_1_ layer to the *n*_2_ medium is larger than the critical angle of total internal reflection (*n*_1_ sin θ_1_ ≥ θ_2_) and κ = 0, then the phase shift due to total internal reflection of X = S or P polarization is Ω_X_ and . Since *r*_01_,Ω_X_, and ϕ are real numbers in eq 2, |*r*|^2^ = 1 and no dip occurs. In other words, the necessary condition for CPA to occur is that there must be even a small amount of absorption in the thin film, i.e., κ ≠ 0.^19^

The necessary conditions for SCCPA using total internal reflection are (in the following expressions, *n*_1_ can be regarded as *n* because κ ≪ 1.)
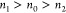
4from ref (4). In order to observe SCCPA using a nearly transparent dielectric material (*q* ≪ 1) for a thin film, the angle of incidence from the substrate to the thin film must be close to π/2. The necessary conditions for SCCPA can be more straightforwardly derived than in the preceding paper^4^ as follows.

(I) Nearly transparent thin films have non-zero absorption

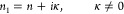

(II) *n*_1_ > *n*_2_ (total internal reflection) must be satisfied.
(III) Reflectance at the interface between the thin film and the substrate must be high because |*r*_01_| = |*r*_12_|*e*^–2*q*^ ≈ *e*^–2*q*^ from eq 3. In other words, incidence angle θ_0_ or θ_1_ must be close to π/2 because of Fresnel’s equations for the reflection coefficients of s- and p-polarized light

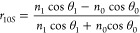
5and
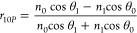
6

(IV) It must be *n*_0_ < *n*_1_ so that light can enter the thin film even at a large incidence angle θ_0_ from the substrate. Therefore, it should be *n*_1_ > *n*_2_ > *n*_0_ or *n*_1_ > *n*_0_ > *n*_2_.
(V) Total reflection at the interface between *n*_1_ and *n*_2_ even at a large incidence angle θ_0_ from *n*_0_

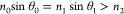
From the above, the condition
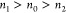
is obtained. For a more detailed discussion of the SCCPA principle, see refs (4)(19),.

## Experimental Section

In order to observe CPA in liquid form, it is necessary to have a highly transparent liquid that separates into two layers without mixing. Therefore, a combination of water and a non-polar organic solvent is a candidate. Thus, we tried to create a film of various types of oil, whose density is smaller than that of water, on the water surface, referring to the literature.^20−22^ We tried toluene as well as oils for oil rotary pumps and a diffusion pump in our laboratory. Among these, silicone oil formed the most stable film, so we prepared silicone oil thin film on water as follows.

**Figure 2:**
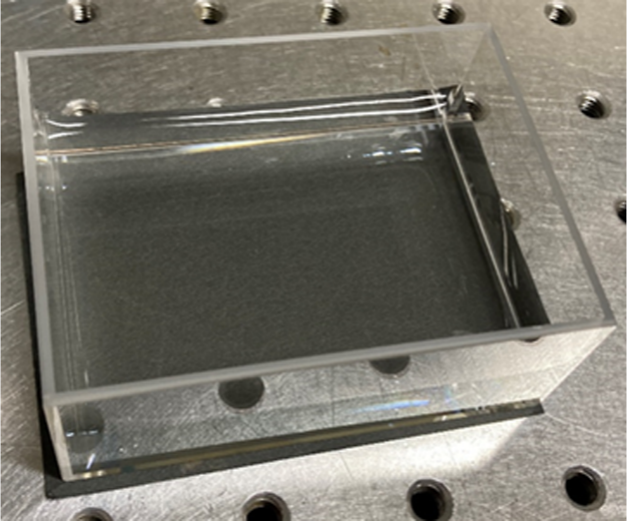
Photograph of oil film on top of water by dropping silicone oil. The distance between the screw holes on the optical breadboard is 25 mm.

A 52 × 72 × 30 mm^3^ quartz cell with a 400–700 nm single-layer anti-reflection coating on the outer surface was used. On top of 80 mL of purified water (refractive index 1.333, high-purity purified water for CPAP, San-Ei Chemical Co., Arao-shi, Kumamoto, Japan) in the cell, silicone oil [KF-96-50CS, polydimethylsiloxane PDMS (C_2_H_6_OSi)_*m*_, polymerization degree m ∼48, molecular weight ∼3500, refractive index 1.402, specific gravity 0.960, kinematic viscosity 50 mm^2^/s, and viscosity 48 mPa·s all at 25 °C, Shin-Etsu Silicone catalog, Shin-Etsu Chemical Co., Tokyo, Japan]. The film thickness was estimated from the numerical calculation of CPA, which will be shown later. The film thickness was adjusted so that a single drop of silicone oil would be sufficient to achieve the desired thickness, since the second drop would separate from the first. Figure 2 shows a photograph of a silicone oil film deposited on top of water.

**Figure 3:**
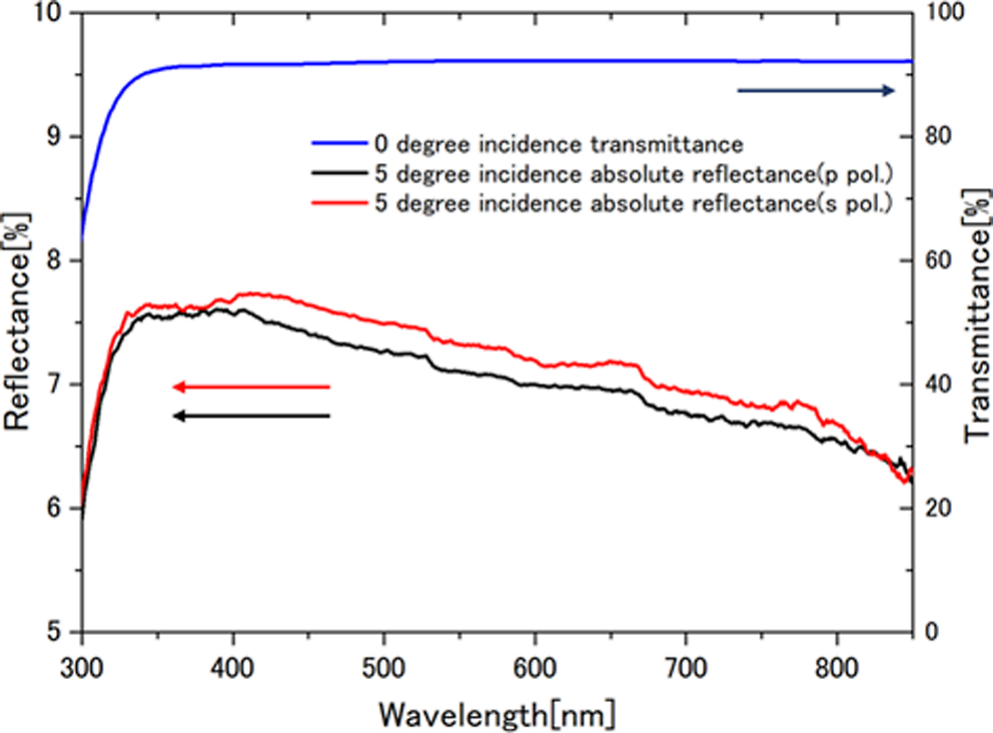
Measured transmittance (normal incidence) and reflectance (5° incidence) of silicone oil in a 1 mm glass cell.

The transmittance of silicone oil at normal incidence and reflectance at 5° incidence is shown in Figure 3. Measurements were made at room temperature with a spectrophotometer (SolidSpec-3700DUV, Shimadzu, Kyoto, Japan). The measurements were made in a borosilicate glass cell with an optical path length of 1 mm, so the transmittance of the oil is flat up to 300 nm, although there is a dip at 300 nm due to the absorption in the cell.

**Figure 4:**
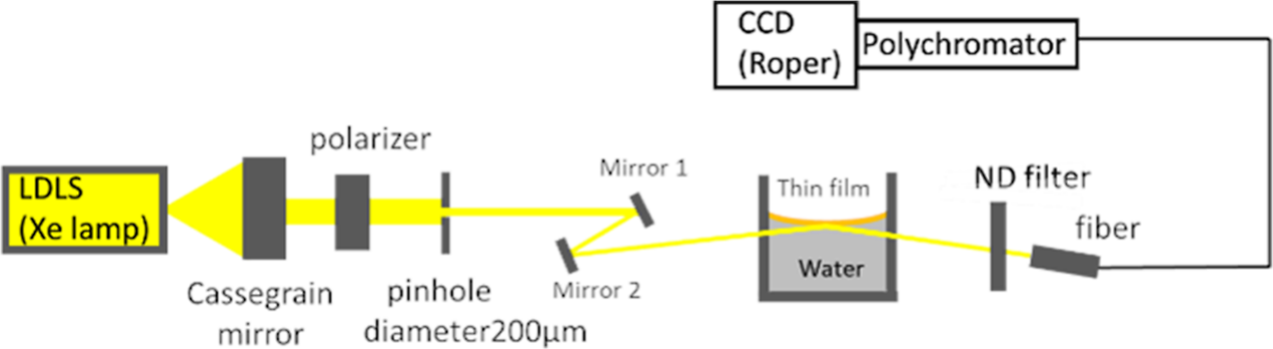
Optical setup for observation of CPA in a liquid sample.

The experiment was performed with the optical system shown in Figure 4. White light from a laser-driven light source (LDLS EQ-99 X, Energetiq Technology, Wilmington, MA, USA) Xe lamp with a specified emission spot size of 100 × 180 μm^2^ was collimated with a Cassegrain mirror and then s- or p-polarized with a Gran-Taylor polarizer. The collimated white light was then passed through a 200 μm pinhole to reduce the beam diameter to 1.37 mm just before it entered the sample. By adjusting Mirror 1 and Mirror 2, the light was incident almost normally into the water from the side of the cell at an angle of incidence close to 90° at the water/oil interface. The multiply reflected light in the sample was emitted from the opposite side of the cell; the light, attenuated by an ND filter, was directed into a fiber bundle and sent to a polychromator (Acton SpectraPro-300i, Acton Research Co., Acton, MA, USA), where it was dispersed with a grating of 150 lines/mm, 500 nm blaze. The spectra in the wavelength range of 350–850 nm were acquired with a wavelength resolution of 5.4 nm at 546 nm by a CCD (liquid nitrogen cooled CCD: PyLoN SPEC-10:2KB, controller: ST-133, Teledyne LeCroy, NY, USA). The imaging array of the CCD is 2048 × 512 , and the pixel size is 13.5 × 13.5 μm^2^.

## Results and Discussion

**Figure 5:**
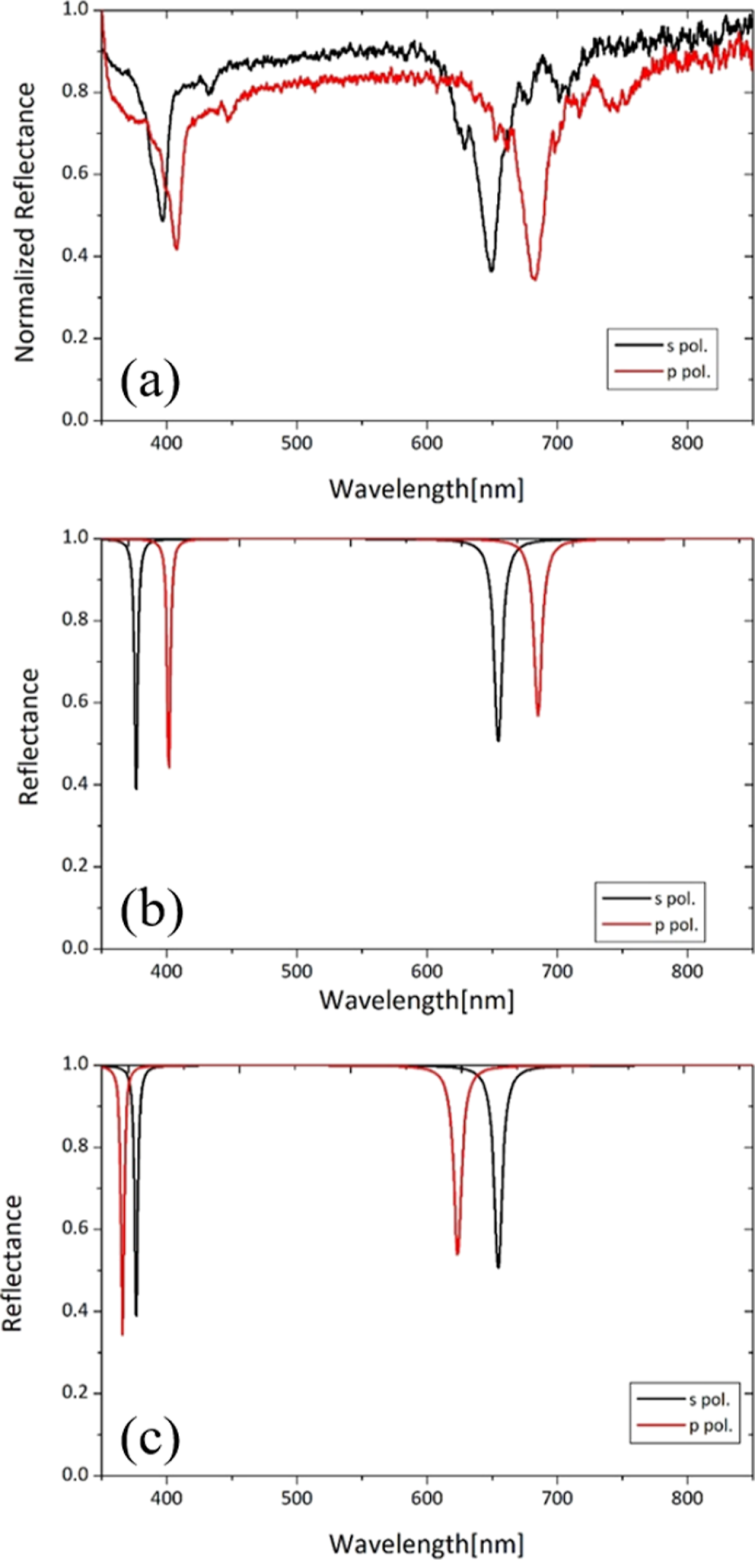
(a) Measured CPA spectra at an angle of incidence 89.5° in air for s-(black) and p-(red) polarization. (b) Calculated CPA spectra at an angle of incidence 89.5° in air with a film thickness of 1020 nm and κ = 0.0001, assuming anisotropic refractive index: *n*_o_ = 1.402 and *n*_e_ = 1.417. (c) Calculated CPA spectra assuming an isotropic refractive index: *n*_o_ = *n*_e_ = 1.402.

The measured and calculated results of s- and p-polarized CPA spectra at an incident angle of 89.5° (incident angle in air θ_air_, as defined in Figure 1, θ_0_ = 89.63° from Appendix A in the Supporting Information) are shown in Figure 5a,b. In the measurement results, reflectance was normalized to a maximum value of 1 by dividing the intensity of signal light reflected by the silicone oil film by the intensity of baseline light passed straight through water without reflection. The reason for normalization is that the angle of the mirror is changed between the signal and baseline measurements, so the optical path changes and the fiber is also moved, so the measurement is not an absolute transmittance measurement. This observation of the reflectance dip in silicone oil on water was the first observation of the CPA phenomenon in a liquid.

The calculations for silicone oil did not take into account the wavelength dependence of the refractive index and extinction coefficient. The refractive index of silicone oil was fixed at 1.402 from the Shin-Etsu Silicone catalog, and the refractive index of water was fixed at 1.333. The thickness *d* and extinction coefficient κ of the silicone oil film (extinction coefficient data was not available) were determined to reproduce the experimental results, with *d* = 1020 nm and κ = 0.0001. In the calculations, as defined in Appendix A in the Supporting Information, anisotropy in the refractive indices for polarization parallel (ordinary) and perpendicular (extraordinary) to the interface^23^ was assumed as *n*_0_(parallel) = 1.402 and *n*_e_(perpendicular) = 1.417, and the results are shown in Figure 5b. As shown in Figure 5c, if the refractive index of the silicone oil film is assumed to be isotropic (*n*_o_ = *n*_e_ = 1.402), the dip positions of s- and p-polarization are opposite to the experimental results. These experimental and calculated results indicate that the refractive index is anisotropic in the direction parallel and perpendicular to the interface (see Appendix B in the Supporting Information). It is a surprising result that an anisotropic refractive index was observed in the liquid compared to the observations of SCCPA in the PVP polymer film on the MgF_2_ substrate in the preceding paper^4^ and in the ITO film on the glass substrate in ref (19), where the dip of p-polarization appears at shorter wavelengths than that of s-polarization and is explained by an isotropic refractive index.

From Figure 5a, silicone oil is transparent visually, but a sharp dip of about 50% can be observed. It may be possible to achieve a phenomenon closer to CPA by optimizing the film thickness or angle of incidence. In Appendix C in the Supporting Information, theoretical conditions for obtaining a 100% dip are searched for by calculation. Since the transparency of the oil is extremely high (κ = 0.0001), the best measure to satisfy the CPA condition |*r*_01_| ≈ *e*^–2*q*^ with *q* = *k*κ *d* cos θ_1_ would be to increase the thickness *d* of the thin film rather than to make |*r*_01_| closer to 1. In this study, we tried a silicone fluid KF-96-50CS with a kinematic viscosity of 50 mm^2^/s, but we were unable to create a stable thin film with a thickness greater than 1 μm. It may be worthwhile to try other silicone oils commercially available that have almost the same physical properties but lower viscosity.

There are possible reasons for incomplete coherent perfect absorption other than the thin film thickness. For example, the finite beam radius, incomplete beam collimation, and phase noise (fluctuations in the thickness or molecular orientations in the film) cause partial beam overlap of the obliquely incident beams, distributed incidence angle, and optical phase fluctuations, respectively, deteriorating the visibility of interference and reducing the depth of the CPA dip.

It is noteworthy that uniaxial refractive index anisotropy was clearly observed in a liquid oil film of about 1 μm thickness on a water surface: the refractive index *n*_o_ = 1.402 for light polarized parallel to the interface (ordinary ray) and *n*_e_ = 1.417 for light polarized perpendicular to the interface (extraordinary ray) as evaluated in the calculation are different by 0.015 as *n*_e_ = 1.01*n*_o_, a difference of only 1%, but the effect is remarkable. The positions where the CPA dip appears for s- and p-polarization on the wavelength axis are reversed, consistent with the experimental results, compared to the case assuming an isotropic refractive index. Why it is so sensitive to minute refractive index anisotropy is discussed quantitatively below (detailed numerical evaluations are provided in Appendix B in the Supporting Information).

The wavelength of the CPA dip is determined by the resonance condition of eq 3: −ϕ_01_ + ϕ_12_ + 2*p* + (2*m* + 1)π = 0 in the preceding paper^4^ and ref (14) In the isotropic case, there is no difference between s- and p-polarization in the phase change  due to light propagation through the thin film, which is evaluated to be 2*p* = 2.706π at λ = 655 nm, the position of the s-polarized dip in Figure 5b,c. Hence, the difference in wavelength of the CPA dip for s- and p-polarization is caused by the difference in phase change due to reflection at both ends of the thin film. The phase change ϕ_01_ (0 → 1) of s- and p-polarization due to reflection at the water–oil interface is almost equal to π because κ is nearly zero, and there is no difference due to polarization. Therefore, the difference in wavelength of the CPA dip of s- and p-polarization is attributed to the difference in phase change due to total reflection at the oil-air interface.

In the isotropic case (*n* = 1.402), the phase change of s- and p-polarization is −0.708π and −0.844π, respectively, and the phase difference between s- and p-polarization is 0.135π. The s-polarization satisfies the resonance condition of *m* = −1 at λ = 655 nm = 1.89 eV (−ϕ_01_ + ϕ_12_ + 2*p* – π = 0 as evaluated in Appendix B) and that of *m* = −2 at λ = 377 nm = 3.29 eV. In other words, adjacent CPA dips are separated by a width of free spectral range (FSR) = 3.29–1.89 = 1.40 eV with a phase difference of 2π. Presuming that the CPA dip energy of p-polarized light shifts from that of s-polarized light by a ratio of the phase difference with s-polarized light to 2π, the dip position of p-polarized light is evaluated to be 1.89 + (3.29 – 1.89) × 0.135π/2π = 1.985 eV = 624 nm, which is in good agreement with Figure 5c (623 nm). In the anisotropic case (*n*_o_ = 1.402, *n*_e_ = 1.417), the phase change of the s-polarization remains the same and that of the p-polarization is −0.832π, so the phase difference between s- and p-polarizations is 0.124π. The expected dip position of p-polarization is 1.89 + (3.29 – 1.89) × 0.124π/2π = 1.977 eV = 627 nm, which shows only a small shift and cannot explain the reversal of the dip position. Therefore, we can conclude that the dip position is reversed by an effect of the phase change 2*p* due to propagation. Let us now evaluate the change in 2*p* for p-polarization when changing from isotropic to anisotropic (2*p* for s-polarization does not change), focusing on the factor *n* cos θ_1_/λ in 2*p* ≈ 4π*nd* cos θ_1_/λ. In the isotropic case, *n* = 1.402 and θ_1_ = 71.95°, so , whereas in the anisotropic case, *n* = *n*_p_ = 1.415 and θ_1_ = α_p_ = 70.37°, so . This means that the resonance wavelength of p-polarization is about 10% longer in the anisotropic case than in the isotropic case, which quantitatively (and perfectly) explains the shift of the dip position (from 623 nm in Figure 5c to 685 nm in Figure 5b) in the calculation results.

The anisotropy of the oil thin film is considered to result from interactions with interfaces. It is possible that the molecular arrangement near the interface with water or air is ordered differently from the bulk,^24^ but the ordering is probably within a few molecular layers or less. In particular, there have been many studies on the ordering of PDMS molecular layers on the water surface using sum frequency generation vibrational spectroscopy,^25−28^ so it is most likely that ordering occurs at the interface of water. Experimental evidence of molecular orientation in a thin silicone oil film on the water surface is provided by Raman spectral measurements in Appendix D. Although no report on the refractive index anisotropy of the PDMS layer at the interface is found, the uniform refractive index anisotropy ratio of 1% over a thickness of 1 μm is considered to be the effective value observed when the extremely large refractive index anisotropy near the water interface is averaged over the entire film thickness.

## Conclusions

The first observation of the CPA phenomenon in a thin film of a liquid was successfully achieved by observing the reflection spectra of a 1020 nm thick silicone oil (PDMS) film on water by collimated white light incident from the water to the oil film under the condition of total reflection at the oil/air interface. The depth of the CPA dip in the reflection spectrum was about 50%. The extinction coefficient of silicone oil was estimated to be κ = 0.0001 from the simulation by the standard Fresnel reflection formula for the monolayer film (eq 2). Due to the high transparency of the oil, more film thickness is needed to obtain a deep dip close to 100%, and it would be promising to try using a PDMS oil with a smaller viscosity. In SCCPA using total reflection, the wavelengths of the s- and p-polarized CPA dips are shifted due to the difference in the phase change of total reflection, but if the refractive index is isotropic, the p-polarized dip appears at a shorter wavelength than the s-polarized dip. However, in the PDMS oil film on water, the wavelength positions of both dips are reversed, clearly indicating that the refractive index of the oil film is anisotropic between in-plane and out-of-plane polarizations. The qualitatively different behavior of the reversal of the order in which the CPA dip appears in the wavelength axis with only 1% refractive index anisotropy indicates that total reflection SCCPA is a sensitive measurement method for detecting the optical anisotropy of thin films.

The visible absorption of water and organic solvents is very weak and difficult to measure by ordinary methods (partly because the reflection is much larger).^29^ However, the weak absorption in the visible region of thin films of transparent materials can be evaluated as the complex refractive index *n* + *i*κ by numerically simulating the incident angle dependence of the SCCPA reflection spectrum or by solving the inverse problem, as the extinction coefficient of silicone oil was evaluated as κ = 0.0001 in the present experiment. In particular, if a deep SCCPA dip can be observed, the approximated value for *q*(≈*k*κ*d* cos θ_1_) can be obtained immediately from eq 4 in the preceding paper,^4^ |*r*_01_| ≈ *e*^–2*q*^. Since only a drop of liquid (1 μL = 1 mm^3^) is sufficient for the thin film preparation in liquid SCCPA, it is considered to be in demand, especially for rare liquids such as ionic liquids, where the preparation of large amounts of samples is difficult.^30^ In addition, since various solute molecules can be dissolved in the liquid, it has the potential to be used for a wide variety of basic and applied research.^4^

## Supporting Information Available

Appendix A: refractive index anisotropy in a thin film; Appendix B: evaluation of phase change due to reflection and propagation of s- and p-polarized light in isotropic and anisotropic thin films; Appendix C: theoretical conditions for obtaining 100% CPA dip; and Appendix D: evidence of molecular orientation in a thin silicone oil film on the water surface by Raman spectral measurements (PDF)

## Notes

The data that support the findings of this study are available from the corresponding author upon reasonable request.

## Abbreviations

CPA: coherent perfect absorption
SCCPA: single-channel coherent perfect absorption
