# Supplementary material for: Observation of Coherent Perfect Absorption in Oil Film on Water Surface and Sensitive Detection of Refractive Index Anisotropy in the Film
Source: Langmuir. 2023 Aug 1;39(32):11357–62. doi: 10.1021/acs.langmuir.3c01189 (PMC10433521; doi:10.1021/acs.langmuir.3c01189)
Supplement: Supplementary file 1 — la3c01189_si_001.pdf [file la3c01189_si_001.pdf]

Supporting Information for

Observation of coherent perfect absorption in oil film on water surface and sensitive detection of refractive index anisotropy in the film

Mayu Hasegawa [1], Junpei Oi [1], Kyohei Yamashita [1], Keisuke Seto [1], Takayoshi Kobayashi [2,3] and Eiji Tokunaga [1,*]

[1]Department of Physics, Faculty of Science, Tokyo University of Science, 1-3 Kagurazaka, Shinjuku-ku, Tokyo 162-8601, Japan; 1221537@ed.tus.ac.jp (M.H.); 1219511@alumni.tus.ac.jp (J.O.); yamashita.k@rs.tus.ac.jp (K.Y.); seto@rs.tus.ac.jp (K.S.)

[2]Advanced Ultrafast Laser Research Center, The University of Electro-Communications, 1-5-1 Chofugaoka, Chofu, Tokyo 182-8585, Japan; kobayashi1901@gmail.com

[3]Department of Electrophysics, National Yang Ming Chiao Tung University, Hsinchu 300, Taiwan

***Correspondence: eiji@rs.tus.ac.jp**

Table of Contents

Appendix A: Refractive Index Anisotropy in a Thin Film 1

Appendix B: Evaluation of phase change due to reflection and propagation of s- and p-polarized light in isotropic and anisotropic thin films 2

Appendix C: Theoretical conditions for obtaining 100% CPA dip 4

Appendix D: Evidence of molecular orientation in a thin silicone oil film on the water surface by Raman spectral measurements 6

Appendix A: Refractive Index Anisotropy in a Thin Film

From the definition of incident angle in air θ_{air} in Figure 1,

$$n_{\text{air}} \sin\left(\frac{\pi}{2} - \theta_{\text{air}}\right) = n_0 \sin\left(\frac{\pi}{2} - \theta_0\right), \qquad \text{(A1)}$$

$$n_0 \sin\theta_0 = n_1 \sin\theta_1 = n_2 \sin\theta_2$$

At the incident angle $\theta_{\text{air}} = 89.5°$ in the present experiment, $n_{\text{air}} = n_2 = 1$, $n_0 = 1.333$, $n_1 = n + i\kappa$, $n = 1.402$, and $\kappa = 0.0001$, so $\theta_0 = 89.63°$ and $\theta_1 = 71.95°$ from $\sin(90° - 89.5°) = 1.333 \sin(90° - \theta_0)$ and $1.333 \sin\theta_0 = 1.402 \sin\theta_1$.

When there is anisotropy in the refractive index between polarization parallel (n_o) and perpendicular (n_e) to the interface (uniaxial anisotropy where the optical axis is perpendicular to the interface), the refractive index is defined as follows.

From the refractive index ellipsoid,

$$\text{s}-\text{polarization:}\quad n_{\text{s}} = n_o \tag{A2}$$

$$\text{p}-\text{polarization:}\quad \frac{1}{{n_{\text{p}}}^2} = \frac{\sin^2\alpha_{\text{p}}}{{n_e}^2} + \frac{\cos^2\alpha_{\text{p}}}{{n_o}^2} \tag{A3}$$

Here, α_{s} and α_{p} is the refraction angle in the thin film for s- and p-polarization. From Snell's law,

$$n_0 \sin\theta_0 = n_{\text{s}} \sin\alpha_{\text{s}} \tag{A4}$$

$$n_0 \sin\theta_0 = n_{\text{p}} \sin\alpha_{\text{p}} \tag{A5}$$

Substituting equation (A3) into equation (A5), we obtain

$$\alpha_{\text{p}} = \arcsin\sqrt{\frac{{n_e}^2{n_0}^2\sin^2\theta_0}{{n_e}^2{n_o}^2 - {n_o}^2{n_0}^2\sin^2\theta_0 + {n_e}^2{n_0}^2\sin^2\theta_0}}\ . \tag{A6}$$

For s-polarization (ordinary ray), since $n_1 = n_o = n_{\text{s}} = 1.402$ using $n_0 = 1.333$, $\theta_0 = 89.63°$ and (A4), $\theta_1 = \alpha_{\text{s}} = 71.95°$. For p-polarization (extraordinary ray), from $n_e = 1.417$, (A5), and (A6), $\alpha_{\text{p}} = 70.37°$ and $n_{\text{p}} = 1.415$.

Appendix B: Evaluation of phase change due to reflection and propagation of s- and p-polarized light in isotropic and anisotropic thin films

Let us consider the incidence of light into a thin film with complex refractive index $n_1 = n + i\kappa$, which is sandwiched between transparent media with refractive index n_0 and n_2 (both real numbers). In this case ($n_0 \to n_1 = n + i\kappa \to n_2$), Snell's law $n_0 \sin\theta_0 = n_1 \sin\theta_1 = (n + i\kappa)\sin\theta_1 = n_2 \sin\theta_2$ holds. We consider such an incidence that the total reflection condition $n_0 \sin\theta_0 \geq n_2$ hold for $n_1 \to n_2$.

[B1] Phase change in s- and p-polarized light on total reflection at $1 \to 2$

$$n_1\cos\theta_1 = \sqrt{n_1^2 - n_1^2\sin^2\theta_1} = \sqrt{(n+i\kappa)^2 - n_0^2\sin^2\theta_0} = \sqrt{n^2 - \kappa^2 - n_0^2\sin^2\theta_0 + i2n\kappa} = \sqrt{u+iv} \qquad (u > 0)$$

$$= x_1 + iy_1 \qquad x_1, y_1 > 0 \tag{A7}$$

Here, $x_1 = \frac{1}{\sqrt{2}}\left(\sqrt{u^2+v^2}+u\right)^{\frac{1}{2}}$ and $y_1 = \frac{1}{\sqrt{2}}\left(\sqrt{u^2+v^2}-u\right)^{\frac{1}{2}}$ while $x_1 = n_1\cos\theta_1$ and $y_1 = 0$ for $\kappa = 0$.

Using the above results, the following equations are obtained:

$$n_2\cos\theta_2 = \sqrt{n_2^2 - n_2^2\sin^2\theta_2} = \sqrt{n_2^2 - n_0^2\sin^2\theta_0} = i\sqrt{n_0^2\sin^2\theta_0 - n_2^2} \tag{A8}$$

$$r_{12\text{S}} = \frac{n_1\cos\theta_1 - n_2\cos\theta_2}{n_1\cos\theta_1 + n_2\cos\theta_2} = \frac{x_1 + iy_1 - i\sqrt{n_0^2\sin^2\theta_0 - n_2^2}}{x_1 + iy_1 + i\sqrt{n_0^2\sin^2\theta_0 - n_2^2}} \tag{A9}$$

When $\kappa = 0$,

$$r_{12\text{S}} = \frac{n_1\cos\theta_1 - i\sqrt{n_1^2\sin^2\theta_1 - n_2^2}}{n_1\cos\theta_1 + i\sqrt{n_1^2\sin^2\theta_1 - n_2^2}} = \frac{\cos\theta_1 - i\sqrt{\sin^2\theta_1 - \left(\frac{n_2}{n_1}\right)^2}}{\cos\theta_1 + i\sqrt{\sin^2\theta_1 - \left(\frac{n_2}{n_1}\right)^2}}\ . \tag{A10}$$

From $r_{12S} = \frac{\cos 71.95° - i\sqrt{\sin^2 71.95° - \left(\frac{1}{1.402}\right)^2}}{\cos 71.95° + i\sqrt{\sin^2 71.95° - \left(\frac{1}{1.402}\right)^2}} = -0.609 - 0.793i$ and $\mathrm{atan}(0.793/0.609) = 0.292\pi$,

$$\phi_{12S} = 0.292\pi - \pi = -0.708\pi \qquad \text{(A11)}$$

$$r_{12P} = \frac{n_2 \cos\theta_1 - n_1 \cos\theta_2}{n_2 \cos\theta_1 + n_1 \cos\theta_2} = \frac{\frac{n_2}{n + i\kappa}(x_1 + iy_1) - \frac{n + i\kappa}{n_2} i\sqrt{n_0^2 \sin^2\theta_0 - n_2^2}}{\frac{n_2}{n + i\kappa}(x_1 + iy_1) + \frac{n + i\kappa}{n_2} i\sqrt{n_0^2 \sin^2\theta_0 - n_2^2}} \qquad \text{(A12)}$$

When $\kappa = 0$,

$$r_{12P} = \frac{(n_2/n_1)^2 \cos\theta_1 - i\sqrt{\sin^2\theta_1 - (n_2/n_1)^2}}{(n_2/n_1)^2 \cos\theta_1 + i\sqrt{\sin^2\theta_1 - (n_2/n_1)^2}} \qquad \text{(A13)}$$

From $r_{12P} = -0.882 - 0.472i$ and $\mathrm{atan}\left(\frac{0.472}{0.882}\right) = 0.156\pi$,

$$\phi_{12P} = 0.156\pi - \pi = -0.844\pi \qquad \text{(A14)}$$

$$\phi_{12S} - \phi_{12P} = 0.135\pi \qquad \text{(A15)}$$

The amount of phase change (absolute value) in total reflection is larger for p-polarized light than for s-polarized light. Since the phase change is negative, p-polarization reduces the optical path length more, i.e., it resonates at shorter wavelengths. Therefore, if the refractive index is isotropic, the dip of p-polarized light will be on the short wavelength side.

In more detail, the dip of s-polarization in Figures 5(b) and 5(c) is at 655 nm. Let us check this resonance condition $-\phi_{01} + \phi_{12} + 2p + (2m+1)\pi = 0$. Phase change due to propagation $2p = 2kn_1 d\cos\theta_1 = \frac{4\pi}{\lambda} n_1 d \cos\theta_1$ is evaluated by substituting $n_1 = n_o = 1.402$, $\theta_1 = 71.95°$, $d = 1020$ nm, and $\lambda = 655$ nm to be $2p = 2.71\pi$, then $2p + \phi_{12S} = 2.00\pi$. Since $-\phi_{01} = -\pi$,

$$-\phi_{01} + \phi_{12} + 2p + (2m+1)\pi = 0 \quad \text{with } m = -1. \qquad \text{(A16)}$$

In the isotropic case, for the same $m = -1$ resonance condition to hold for p-polarized light, the resonance wavelength λ is shortened so that $2p$ becomes larger, since ϕ_{12P} is a larger negative phase change than ϕ_{12S}.

[B2] Phase change of s- and p-polarization due to reflection at 1 → 0

$$1 \to 0 \quad r_{10S} = \frac{n_1 \cos\theta_1 - n_0 \cos\theta_0}{n_1 \cos\theta_1 + n_0 \cos\theta_0} = \frac{x_1 + iy_1 - n_0 \cos\theta_0}{x_1 + iy_1 + n_0 \cos\theta_0}$$

$$= \frac{\sqrt{n^2 - \kappa^2 - n_0^2 \sin^2\theta_0 + i2n\kappa} - n_0 \cos\theta_0}{\sqrt{n^2 - \kappa^2 - n_0^2 \sin^2\theta_0 + i2n\kappa} + n_0 \cos\theta_0} \qquad \text{(A17)}$$

Using $n_0 = 1.333$, $n_1 = n + i\kappa$, $n = 1.402$, $\kappa = 0.0001$, $n_2 = 1$, $\theta_0 = 89.63°$, and $\theta_1 = 71.95°$,

$$r_{10S} = 0.961(1 + 0.0000297i) = 0.961e^{0.0000297i} \qquad \text{(A18)}$$

$$1 \to 0 \quad r_{10P} = \frac{n_0 \cos\theta_1 - n_1 \cos\theta_0}{n_0 \cos\theta_1 + n_1 \cos\theta_0} = \frac{\frac{n_0}{n_1} n_1 \cos\theta_1 - n_1 \cos\theta_0}{\frac{n_0}{n_1} n_1 \cos\theta_1 + n_1 \cos\theta_0} = \frac{\frac{n_0}{n + i\kappa}(x_1 + iy_1) - (n + i\kappa)\cos\theta_0}{\frac{n_0}{n + i\kappa}(x_1 + iy_1) + (n + i\kappa)\cos\theta_0},$$

$$= \frac{n_0\sqrt{n^2 - \kappa^2 - n_0^2 \sin^2\theta_0 + i2n\kappa} - (n + i\kappa)^2 \cos\theta_0}{n_0\sqrt{n^2 - \kappa^2 - n_0^2 \sin^2\theta_0 + i2n\kappa} + (n + i\kappa)^2 \cos\theta_0} \qquad \text{(A19)}$$

This is evaluated with the same experimental values above as

$$r_{10P} = 0.957(1 + 0.0000266i) = 0.957e^{0.0000266i} \qquad \text{(A20)}$$

Thus, the effect of the phase change due to reflection at 1→0 is negligible. Similarly, the reflection from 0→1 adds π to the phase change at 1→0, and no difference occurs between s- and p-polarization.

[B3] Mechanism of the red shift in CPA dip of p-polarized light due to anisotropic refractive index

Let us evaluate the phase change of p-polarization due to total reflection by Eq. (A13) for the anisotropic case to be compared with the result $\phi_{12P} = -0.844\pi$ in (A14) for the isotropic case.

Substituting $n_1 = n_\mathrm{p} = 1.415$ and $\theta_1 = \alpha_p = 70.37°$, we obtain $r_{12\mathrm{P}} = -0.865 - 0.503\mathrm{i}$ and $\mathrm{atan}\left(\frac{0.503}{0.865}\right) = 0.168\pi$, thus

$$\phi_{12\mathrm{P}} = 0.168\pi - \pi = -0.832\pi \qquad \text{(A21)}$$

As discussed in the main text, this change in $\phi_{12\mathrm{P}}$ does not reverse the CPA dip wavelength. Therefore, we evaluate below the effect of the phase change $2p = 2kn_1 d\cos\theta_1 = \frac{4\pi}{\lambda} n_1 d\cos\theta_1$ due to the light propagation on the CPA dip. We pay attention to the factor of $\frac{n_1\cos\theta_1}{\lambda}$ in the formula for $2p$ for the anisotropic refractive index of $n_o = 1.402$ and $n_e = 1.417$.

For s-polarization in both isotropic and anisotropic cases and p-polarization in the isotropic case, substituting $n_1 = n_\mathrm{s} = n_o = 1.402$ and $\theta_1 = \alpha_s = 71.95°$,

$$\frac{n_1\cos\theta_1}{\lambda} = \frac{0.4345}{\lambda} \qquad \text{(A22)}$$

For p-polarization in the anisotropic case, substituting $n_1 = n_\mathrm{p} = 1.415$ and $\theta_1 = \alpha_\mathrm{p} = 70.37°$,

$$\frac{n_1\cos\theta_1}{\lambda} = \frac{0.4754}{\lambda} \qquad \text{(A23)}$$

Equations (A22) and (A23) tell us that the resonance wavelength λ of p-polarized light for the anisotropic case should be 10% longer than that for the isotropic case, quantatively explaining the shift of the dip position (from 623 nm in Figure 5(c) to 685 nm in Figure 5(b)) in the calculation results.

Appendix C: Theoretical conditions for obtaining 100% CPA dip

The approximate condition of CPA (100 % dip) is given by Eq. (4) in Ref.[4] as

$|r_{01}| \approx e^{-2q}$ with $q \approx k\kappa d\cos\theta_1$ and $|r_{01}| = \left|\frac{n_1\cos\theta_1 - n_0\cos\theta_0}{n_1\cos\theta_1 + n_0\cos\theta_0}\right|$.

This tells us that if the present experimental condition is $|r_{01}| < e^{-2q}$, then the CPA condition (100 % dip) can be obtained by either increasing the angle of incidence θ_0 (or θ_air) closer to 90 degrees, or by increasing q without changing the angle of incidence, i.e., by increasing κd by keeping $|r_{01}|$ and $\cos\theta_1$ constant. Since the incomplete collimation of the probe beam does not allow for controlling the incidence angle in air with a precision of 0.1 degree, a practical solution is to increase the film thickness. Figures S1, S2, and S3 show how the CPA dip theoretically depends on θ_air, κ, and d, respectively, if they are changed from the present experimental condition of $\theta_\mathrm{air} = 89.5°, \kappa = 0.0001$, and $d = 1020$ nm.

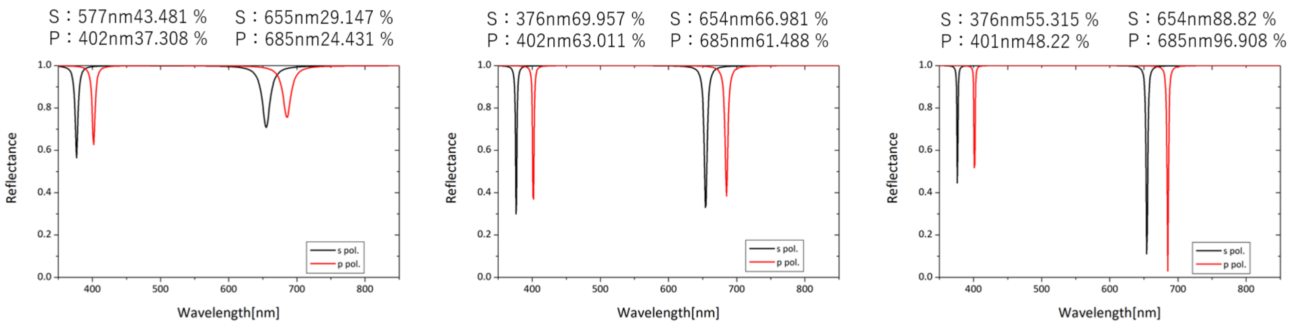

89.0° $d = 1020$ nm $\kappa = 0.0001$ 89.7° $d = 1020$ nm $\kappa = 0.0001$ 89.9° $d = 1020$ nm $\kappa = 0.0001$

Figure S1. Dependence of calculated CPA spectra on θ_{air}, the angle of incidence in air.

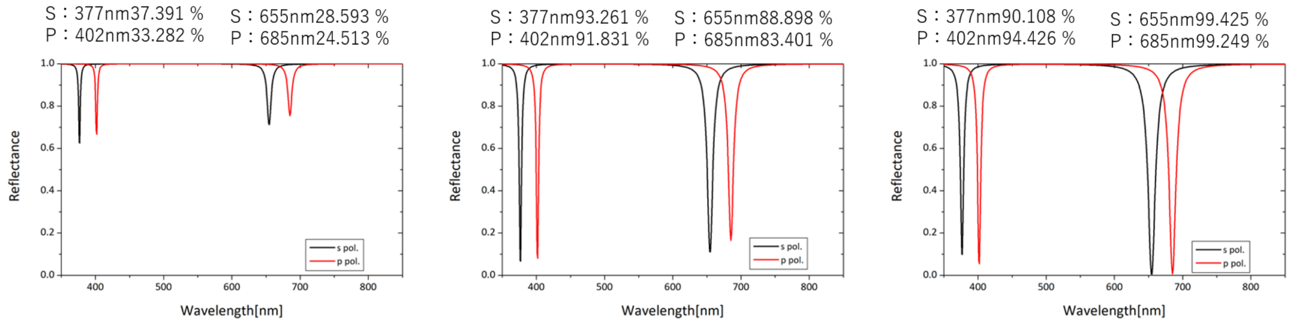

89.5° $d = 1020$ nm $\kappa = 0.00005$ 89.5° $d = 1020$ nm $\kappa = 0.0003$ 89.5° $d = 1020$ nm $\kappa = 0.0006$

Figure S2. Dependence of calculated CPA spectra on κ.

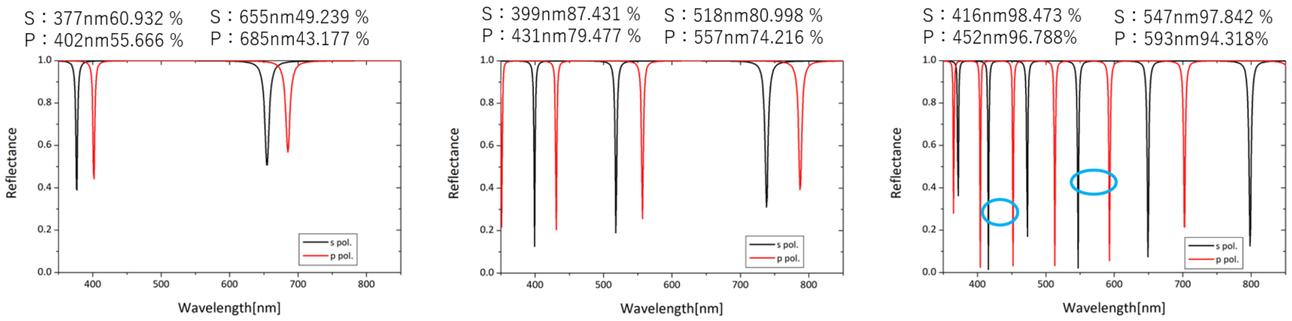

89.5° $d = 1020$ nm $\kappa = 0.0001$ 89.5° $d = 2000$ nm $\kappa = 0.0001$ 89.5° $d = 4000$ nm $\kappa = 0.0001$

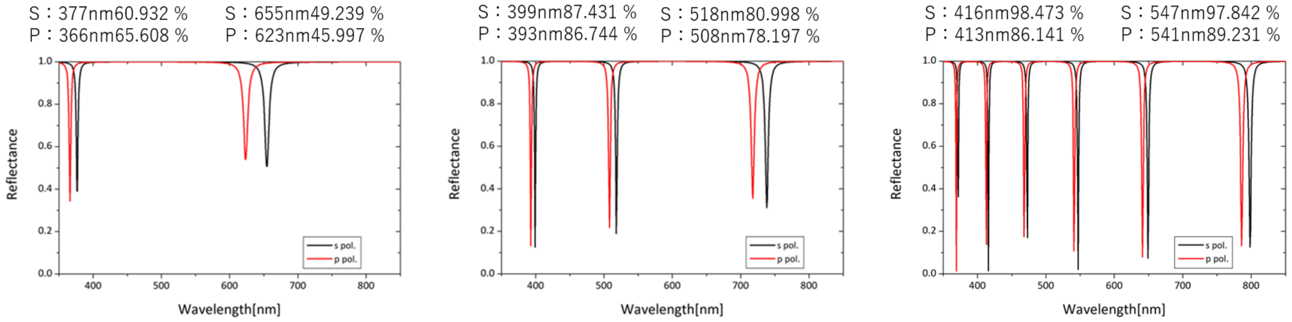

Figure S3. Dependence of calculated CPA spectra on the film thickness d. Top: $n_o = 1.402$ and $n_e = 1.417$. Bottom: $n_o = n_e = 1.402$. Top and bottom left: the same as Figs. 5(b) and 5(c). Note that the wavelengths at the

dip positions of p-polarization are 10% longer in the Top spectra (anisotropic) than in the Bottom spectra (isotropic) as shown in Equations (A22) and (A23).

Although both r_{01} and q depend on the incidence angle θ_0, q is almost independent of the incidence angle if θ_0 is very close to $\frac{\pi}{2}$, and $|r_{01}| \rightarrow 1$ as $\theta_0 \rightarrow \pi/2$, from the following analysis. If θ_0 and θ_1 are both close to $\frac{\pi}{2}$, then $\theta_0 = \frac{\pi}{2} - \delta_0$ and $\theta_1 = \frac{\pi}{2} - \delta_1$ with $\delta_0 \ll 1$ and $\delta_1 \ll 1$. Then

$$n_0 \sin\theta_0 = n_1 \sin\theta_1 \approx n \sin\theta_1 \quad n_0 < n$$

$$n_0 \sin\left(\frac{\pi}{2} - \delta_0\right) = n_1 \sin\left(\frac{\pi}{2} - \delta_1\right) \approx n \sin\left(\frac{\pi}{2} - \delta_1\right)$$

$$n_0 \cos\delta_0 = n_1 \cos\delta_1 \approx n \cos\delta_1$$

$$n_0\left(1 - \frac{\delta_0^2}{2}\right) \approx n\left(1 - \frac{\delta_1^2}{2}\right)$$

$$\delta_1 \approx \left[2\left(1 - \frac{n_0}{n}\right) + \frac{n_0}{n}\delta_0^2\right]^{\frac{1}{2}} \approx \left[2\left(\frac{n - n_0}{n}\right)\right]^{\frac{1}{2}}\left[1 + \left(\frac{n_0}{n - n_0}\right)\frac{\delta_0^2}{4}\right]$$

$$\cos\theta_0 = \cos\left(\frac{\pi}{2} - \delta_0\right) = \sin\delta_0$$

$$\cos\theta_1 = \cos\left(\frac{\pi}{2} - \delta_1\right) = \sin\delta_1$$

Therefore, if second order terms with respect to δ_0 are neglected,

$$q \approx k\kappa d\cos\theta_1 \approx k\kappa d\sin\delta_1 \approx k\kappa d\delta_1 \approx k\kappa d\left[2\left(\frac{n-n_0}{n}\right)\right]^{\frac{1}{2}}\left[1 + \left(\frac{n_0}{n-n_0}\right)\frac{\delta_0^2}{4}\right] \approx k\kappa d\left[2\left(\frac{n-n_0}{n}\right)\right]^{\frac{1}{2}} \quad (\delta_0 \ll 1) \qquad \text{(A24)}$$

$$|r_{10s}| = \left|\frac{n_1\cos\theta_1 - n_0\cos\theta_0}{n_1\cos\theta_1 + n_0\cos\theta_0}\right| \approx \left|\frac{n\sin\delta_1 - n_0\sin\delta_0}{n\sin\delta_1 + n_0\sin\delta_0}\right| \approx \left|\frac{n\delta_1 - n_0\delta_0}{n\delta_1 + n_0\delta_0}\right| \approx \left|\frac{n\left[2\left(\frac{n-n_0}{n}\right)\right]^{\frac{1}{2}} - n_0\delta_0}{n\left[2\left(\frac{n-n_0}{n}\right)\right]^{\frac{1}{2}} + n_0\delta_0}\right| \quad (\delta_0 \ll 1) \qquad \text{(A25)}$$

Appendix D: Evidence of molecular orientation in a thin silicone oil film on the water surface by Raman spectral measurements

[D1] Experimental Procedure

Raman spectra were measured in backscattering geometry with an NRS-3200 laser Raman microscope (JASCO, Tokyo, Japan) equipped with a cooled CCD camera (−50 C°). The objective lens used was UMPLFL ×20, NA = 0.46 (Olympus, Tokyo, Japan). The polarization directions of the excitation light and the observed Raman scattering light were both parallel to the liquid surface. The excitation wavelength was 532.3 nm, and the excitation power was 36 mW. The wavenumber resolution was set to 4 cm^{-1}. The Raman spectrum of bulk silicone oil was obtained by accumulating 30 measurements for an exposure time of 10 s. A silicone oil film about 1-μm thick on the water surface was obtained from 6.5 μL of silicone oil spread on the surface of the water

in a petri dish with an inner diameter of 92 mm. The Raman spectrum of the oil film was obtained by accumulating 3 measurements for an exposure time of 100 s.

[D2] Raman Spectra

Bulk

Figure S4 shows the Raman spectrum of the bulk silicone oil.

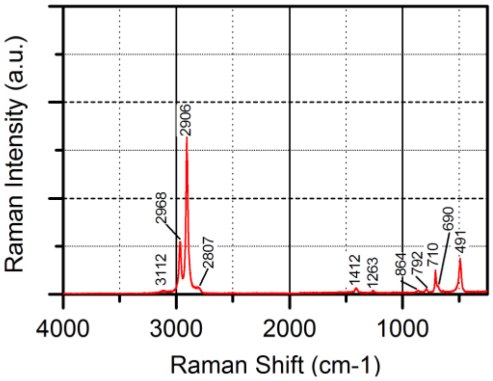

Figure S4. Raman spectrum of the bulk silicone oil.

It is known that the Raman spectra of silicone oils (polydimethylsiloxane (PDMS)) vary with the degree of polymerization [A1]. The obtained spectrum is nearly the same as that with an average molecular weight of 58100 shown in the literature [A1]: The peaks of the vibration bands at 2968, 2906, 1412, 1263, 864, 792, 710, 690, and 491 cm^{-1} are respectively assigned to C-H stretching, C-H stretching, C-H bending, C-H bending, CH_3 rocking, C-Si-C stretching, C-Si-C stretching, CH_3 rocking, and Si-O stretching modes. The vibrational modes of the same chemical bonds have different wavenumbers because of the different relative vibrational phases among the chemical bonds. Here, the symmetry of the PDMS local structure (O-Si(CH_3)$_2$-O) belongs to the C_{2v} point group.

Silicone oil film on the water surface

Figure S5 shows the Raman spectrum of the silicone oil film on the water surface.

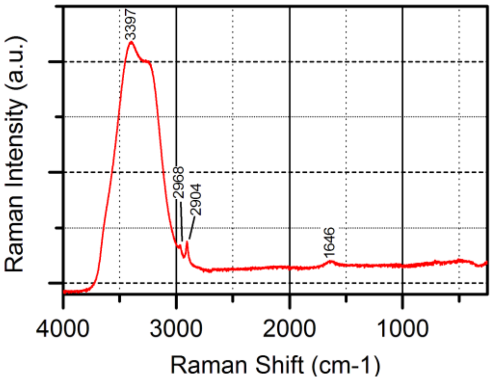

Figure S5. Raman spectrum of the silicone oil film on the water surface.

The bands with peaks at 3397 and 1646 cm^{-1} are water bands. The bands for C-H stretching modes of silicone oil were observed, but the other bands were weak. Figure S6 shows a spectrum magnified so that the 2906-cm^{-1} band intensity in Figure S5 is the same as that in Figure S4.

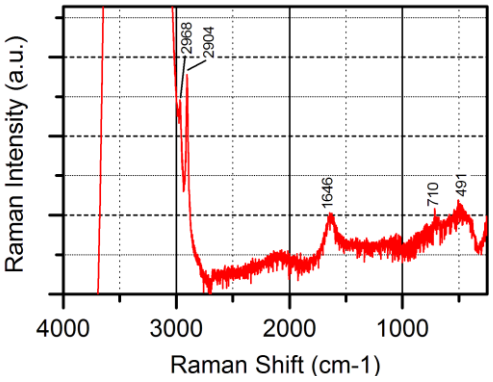

Figure S6. Magnified spectrum with the intensity of the 2906-cm^{-1} band in Figure S5 being the same magnitude as that in Figure S4.

The 491- and 710-cm^{-1} bands were observed with very low S/N, but the relative intensity ratios are clearly lower than those in bulk. The ratio of the 491-cm^{-1} band area to the 2906-cm^{-1} band area in the bulk oil was 0.17. On the other hand, the ratio for the film was 0.049, less than 1/3 of that for the bulk. The 2906-cm^{-1} band area was obtained by fitting the water band as a baseline with a five-band model [A2] and fitting the C-H stretching modes with five Voigt functions. A shoulder appeared on the high wavenumber side of the 491-cm^{-1} band, so its band area was obtained by fitting with two Voigt functions with a straight line as the baseline.

In general, the intensity/polarization of the excitation light and the intensity/polarization of the Raman scattered light are related by the Raman tensor, and the difference in the Raman band intensity ratio in the bulk and the film suggests that the molecules are oriented in the liquid film [A3, A4]. In other words, in the bulk, where the molecules are not oriented, the Raman bands are observed as contributions from the spatially averaged Raman tensor. In a system with oriented molecules, the contribution of a specific tensor component is larger, so the Raman band intensity ratio is different from that in the bulk, reflecting the difference in the magnitude of the tensor component among vibrational modes. In the present experiment, therefore, it is clear that the molecules are oriented in the silicone film on water.

In the following, let us consider whether one can determine the specific molecular orientation from the present Raman spectra. Since the symmetry of the Raman tensor is determined by the symmetry of the vibrational modes, the molecular orientation can be estimated from spectra measured in backscattering geometry, depending on the symmetry of the molecule [A4]. However, the local symmetry of PDMS is the C_{2v} group, and the irreducible representation to which the observed 710- and 491-cm^{-1}-bands in the film belong is identical to A_1. That of the 2968-cm^{-1}- band is an overlap of A_1, A_2, B_1, and B_2, while that of the 2906-cm^{-1}-band is A_1 and B_1. In other words, the symmetry of the Raman tensor is indistinguishable for all the observed modes. Therefore, determining the orientation requires a ratio of the magnitudes of the tensor components, which is currently difficult to do.

[A1] Jayes, L.; Hard, A. P.; Séné, C.; Parker, S. F.; Jayasooriya, U. A. Vibrational Spectroscopic Analysis of Silicones: A Fourier Transform-Raman and Inelastic Neutron Scattering Investigation. *Anal. Chem*. 2003, 75(4), 742-746. DOI: https://doi.org/10.1021/ac026012f

[A2] Baschenko, S. M.; Marchenko, L. S. On Raman spectra of water, its structure and dependence on temperature. *Semicond. Phys. Quantum Electron. Optoelectron.* 2011, **14**(1), 77-79. DOI: https://doi.org/10.15407/spqeo14.01.077

[A3] Basova, T. V.; Kolesov, B. A. Raman polarization studies of the orientation of molecular thin films, *Thin Solid Films* 1998, **325**(1-2), 140-144. DOI: https://doi.org/10.1016/S0040-6090(98)00485-4

[A4] Seto, K.; Furukawa, Y. Study on solid structure of pentacene thin films using Raman imaging. *J. Raman Spectrosc.*, 2012, **43**(12), 2015-2019. DOI: https://doi.org/10.1002/jrs.4090
